# 

**Published:** 1895-05

**Authors:** 


### PERSONAL.

Dr. M. W. Drake is an applicant for the position of City Veterinarian to the Department of Public Safety under the new administration. He is a graduate of the American Veterinary College, but is not actively identified with any of the leading veterinary associations, local or State.

Veterinarians Gadsden, of Philadelphia, Pa.; Parker, of Haverhill, Mass., and Wright, of Burlington, Vt., conducted the practical examination of the class of '95 at the Veterinary Department of McGill University, Montreal, Canada.

Dr. Richard Ebbitt, formerly of Omaha, Neb., has been transferred to Indianapolis by the Meat Inspection Department of the Bureau of Animal Industry.

Dr. W. S. Devoe was hastily dispatched by the Bureau of Animal Industry to investigate the suspected outbreak of contagious pleuro-pneumonia in Kansas. His long experience and acute perceptions, trained by many former years in this line of investigation, soon dissipated all question as to the existence of this disease, and once again made us all breathe easier.

Dr. John R. Hart, the present City Veterinarian of Philadelphia, has held the position for many years. Under his faithful services the position has grown in importance and worth, and the compensation increased from time to time, until the present salary has become an attractive one. We are quite sure that any consideration of his removal does not emanate from any lack of discharge of duty by the present incumbent, for a more conscientious, faithful and earnest worker fills no public position of this kind in this country. We sincerely trust that if a change is anticipated that this position will be placed absolutely under the civil service, and that its attainment will only be secured by a thorough examination, by a committee of experts, on a similar plan as pertained to the recent choice of a chief and assistant bacteriologist to our City Board of Health.

Dr. W. B. E. Miller, Meat Inspector for the city of Camden, N. J., has been succeeded by the appointment of Dr. E. H. Landes. Another move on the political checker board is again recorded. When will our professional friends learn the lesson that follows in the train of uncertainty of these positions when so made? When will the merit system prevail and end this disgraceful farce among an intelligent, thinking people?

Professor S. J. J. Harger has accepted the direction of the veterinary department of the new journal, *Coaching*, published at Philadelphia, a paper devoted to the interests of the rider, driver, and all adjunct interests of coaching.

Dr. E. Mayhew Michener, formerly of Colmar, Pa., has removed to North Wales, Pa.

Dr. James A. Waugh, Veterinarian to the Duquesne Kennel Club, acted in a similar capacity to the recent bench show, held at Pittsburg in April, under the direction and management of the above club.

